# Genome-wide association study of trypanosome prevalence and morphometric traits in purebred and crossbred Baoulé cattle of Burkina Faso

**DOI:** 10.1371/journal.pone.0255089

**Published:** 2021-08-05

**Authors:** Bernadette Yougbaré, Albert Soudré, Dominique Ouédraogo, Bienvenue L. Zoma, Arnaud S. R. Tapsoba, Moumouni Sanou, Salifou Ouédraogo-Koné, Pamela A. Burger, Maria Wurzinger, Negar Khayatzadeh, Hamidou H. Tamboura, Okeyo Ally Mwai, Amadou Traoré, Johann Sölkner, Gábor Mészáros

**Affiliations:** 1 Department of Sustainable Agricultural Systems, University of Natural Resources and Life Sciences, Vienna (BOKU), Vienna, Austria; 2 Institut de l’Environnement et de Recherches Agricoles (INERA), Ouagadougou, Burkina Faso; 3 Unité de Formation et de Recherche en Sciences et Techniques, Université Norbert Zongo de Koudougou, Koudougou, Burkina Faso; 4 Institut du Développement Rural, Université Nazi Boni de Bobo-Dioulasso, Bobo-Dioulasso, Burkina Faso; 5 Department of Integrative Biology and Evolution, University of Veterinary Medicine Vienna (Vetmeduni), Vienna, Austria; 6 International Livestock Research Institute (ILRI), Nairobi, Kenya; Institute of Farm Animal Genetics, GERMANY

## Abstract

In this study, single-SNP GWAS analyses were conducted to find regions affecting tolerance against trypanosomosis and morphometrics traits in purebred and crossbred Baoulé cattle of Burkina Faso. The trypanosomosis status (positive and negative) and a wide set of morphological traits were recorded for purebred Baoulé and crossbred Zebu x Baoulé cattle, and genotyped with the Illumina Bovine SNP50 BeadChip. After quality control, 36,203 SNPs and 619 animals including 343 purebred Baoulé and 279 crossbreds were used for the GWAS analyses. Several important genes were found that can influence morphological parameters. Although there were no genes identified with a reported strong connection to size traits, many of them were previously identified in various growth-related studies. A re-occurring theme for the genes residing in the regions identified by the most significant SNPs was pleiotropic effect on growth of the body and the cardiovascular system. Regarding trypanosomosis tolerance, two potentially important regions were identified in purebred Baoulé on chromosomes 16 and 24, containing the CFH, CRBN, TRNT1 and, IL5RA genes, and one additional genomic region in Baoulé, x Zebu crossbreds on chromosome 5, containing MGAT4C and NTS. Almost all of these regions and genes were previously related to the trait of interest, while the CRBN gene was to our knowledge presented in the context of trypanosomiasis tolerance for the first time.

## Introduction

In this study we focus on the discovery of regions affecting tolerance against trypanosomosis and morphometric traits in purebred Baoulé and crossbred Zebu x Baoulé cattle of Burkina Faso. Baoulé cattle (*Bos taurus*), locally called Lobi cattle, is an important taurine population located in the Southwest West of Burkina Faso, a region known to be tsetse challenged [1, 2]. This cattle breed has existed in the region for a long time and acquired an immunological phenomenon (trypanotolerance) with a likely genetic basis. These animals have a capacity to rid themselves of trypanosome parasites and maintain low parasitemia [3, 4]. Trypanotolerance is presumed to have arisen through natural selection acting on cattle exposed to trypanosome infection. Trypanotolerance is a heritable trait associated with ability to control anemia, a major clinical sign during disease development [5]. Thus, trypanotolerant animals have been introduced in other tsetse affected countries of Africa in order to exploit their genetic advantage. Unfortunately, most of these taurine cattle types are very small in body size, with height at withers of less than 100 cm. This makes them much less useful for ploughing purposes than the bigger but susceptible Zebu populations found nearby.

To make up for the small body size of the trypanotolerant breeds, they often have been crossed to other, larger breeds. These breeds, like the humped Zebu populations started inhabiting these regions only with the development of veterinary prophylaxis and the destruction of the tsetse habitat through widespread deforestation [6]. As the Zebu cattle have been introduced by migrants Fulani in the region just recently, they presumably lack trypanotolerant characteristics. This was also supported by the proportionally higher death rate compared to local cattle [7] presumable in a substantial part due to lack of positive reaction to trypanosomiasis infections.

In particular, in this study we were interested in these crossbred animals from a genetic point of view, as they represent an unknown genetic base with potential trypanotolerance features from the Baoulé side and increased size and other desired *Bos indicus* features from the zebu side. The aim of the paper is to acquire an insight into the genetic mechanisms underlying trypanotolerance and morphometric traits in Baoulé cattle and their crossbreds with Zebu.

## Materials and methods

The trypanosomosis status and a wide set of morphological traits was recorded for 646 animals including 360 purebred Baoulé and 286 crossbred Zebu x Baoulé cattle from the Southwest of Burkina Faso. EDTA blood samples were collected during health monitoring for trypanosome infection in cattle in Burkina Faso by the Institut de l’Environnement et de Recherches Agricoles (INERA), BP 8645 Ouagadougou, Burkina Faso within the framework of the project #120 ‘Local cattle breeds of Burkina Faso–characterization and sustainable utilization’ approved and funded by Austrian Partnership Programme in Higher Education and Research for Development (APPEAR), in cooperation with the Unité de Formation et de Recherche en Sciences et Techniques from the Université Norbert Zongo de Koudougou, and the Institut du Développement Rural, Université Nazi Boni de Bobo-Dioulasso, Burkina Faso. The indirect Elisa has been used for the diagnosis of positive or negative trypanosomosis infection from blood samples [8]. The phenotypic measurements included 10 qualitative traits (Table 1) and 25 quantitative traits (Table 2). The methodology of measurements of quantitative traits followed the FAO guidelines on phenotypic characterization of animal genetic resources [9].

**Table 1:** List of measured qualitative traits with the different levels of characterization.

Traits (9)	Level
Color of muzzle	2 levels: Pigment/No pigment
Eyelid pigmentation	2 levels: Black/white
Color of the hoof	2 levels: Pigment/No pigment
Color of extremity	2 levels: Charred/Faded
Speckled coat	2 levels: No/Yes
Dewlap size	2 levels: Well-developed/ Poorly developed
Ears’ position	2 levels: Vertical upwards /Horizontal
White variegations	2 levels: Irregular/Lateral color
Development of udder	2 levels: Well-developed/ Poorly developed

**Table 2:** List of measured quantitative traits.

Head and horn measurements (n = 11)	Body measurements (n = 14)
cranial length, head width, head length, cranial width, facial length, facial width, muzzle circumference, distance between horn tips, distance between horn bases, horn length, earn length	height at withers, chest girth, height at sacrum, body length, length of scapula ischium, hip width, ischium width, tail length, chest depth, shoulder width, chest width, teat length, basin length, height of the hump

The genotype data from the Illumina Bovine SNP50 BeadChip were available for all animals. Quality control of the data was performed with PLINK 1.9 [10]. The dataset was cleaned using standard quality control to exclude non-autosomal SNPs as well as SNPs with minor allele frequency lower than 0.01, those with a call rate of <95% and those that deviated from Hardy Weinberg equilibrium with Fisher’s exact test with P-value < 10E-6. Animals with a call rate <90% were excluded. After applying quality control, 38,322 SNPs and 619 animals including 340 purebred Baoulé and 279 Baoulé and Zebu crossbreds were used for the GWAS analyses.

For the association study a univariate linear mixed model for marker association tests was fitted with a single phenotype as:

y=μ+Xβ+sex+age+ε

where y was the vector of phenotypes; μ was the intercept; X was a vector of SNP genotypes; β was the effect size of the SNP; and ε is a vector of errors. In addition, the *sex* of the individual (male, female) and the continuous variable of *age* were considered as covariates by integrating the covariance of sex, age and race in this model. For the age, the average was 3.76 years (standard deviation of 2.86), with a maximum and minimum of 18 and 0.08 years, respectively. As for the sex, the number of females was 399 and the number of males was 220.

Single-SNP associations based on the genome-wide efficient mixed model association algorithm were performed using GEMMA [11]. The genomic relationship matrix was used to account for population structure. The two significance thresholds used in the evaluation of the results were an arbitrary indicative threshold of–log_10_(p) = 5, and the threshold based on the Bonferroni correction with a base of p = 0.05, at–log_10_(p) = 5.86. The region within ±0.5 Mb of a SNP above the threshold was declared as significant when searching for any associated genes. The significant regions were checked for genes based on the ARS UCD1.2 *Bos Taurus* Genome Assembly on the NCBI database.

## Results and discussion

From the wide range of measured traits we intend to focus on those that describe the potential morphological differences between taurine and zebu cattle. From the qualitative traits these were ear shape and dewlap size, both typical and easily recognizable characteristics of the zebu cattle. From the quantitative traits these were the general body size measurements, such as Height at withers, body length, chest width, depth and girth. The differences in head size were analysed via the head length and width, cranial length and width, as well as the length of the ear. Furthermore, we searched for genomic regions with a possible influence on trypanosomiasis tolerance. By associating the results of indirect Elisa for trypanosomosis infection and genotypes of purebred Baoulé cattle, as well as their crosses we identified genomic regions with significant SNPs and their underlying genes.

### Genome-wide association for morphological traits—qualitative traits

The GWAS analysis of qualitative traits with the fixed effects of sex and age allowed identifying regions in a number of chromosomes responsible for these traits (Table 3).

**Table 3:** Chromosomes with significant effects for qualitative traits.

Traits	Chromosome number	Number of analyzed individuals
Ears’ position	1,2,5,8,9,10,11,14,16,17,18,20,21,22,24,25,28	198
Dewlap size	2, 12	198

Both, the elongated floppy ears as well as the increased navel and dewlap size in Zebu are a consequence of increased thermoregulatory mechanisms via excess skin.

**Fig 1:**
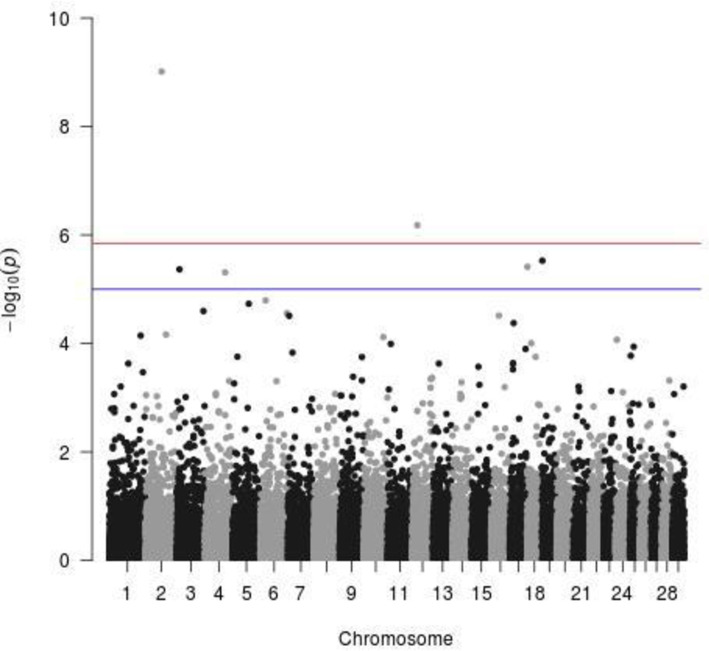
Significant SNPs for the dewlap size trait (blue line = indicative threshold–log10(p) = 5; red line = Bonferroni threshold–log10(p) = 5.86).

**Fig 2:**
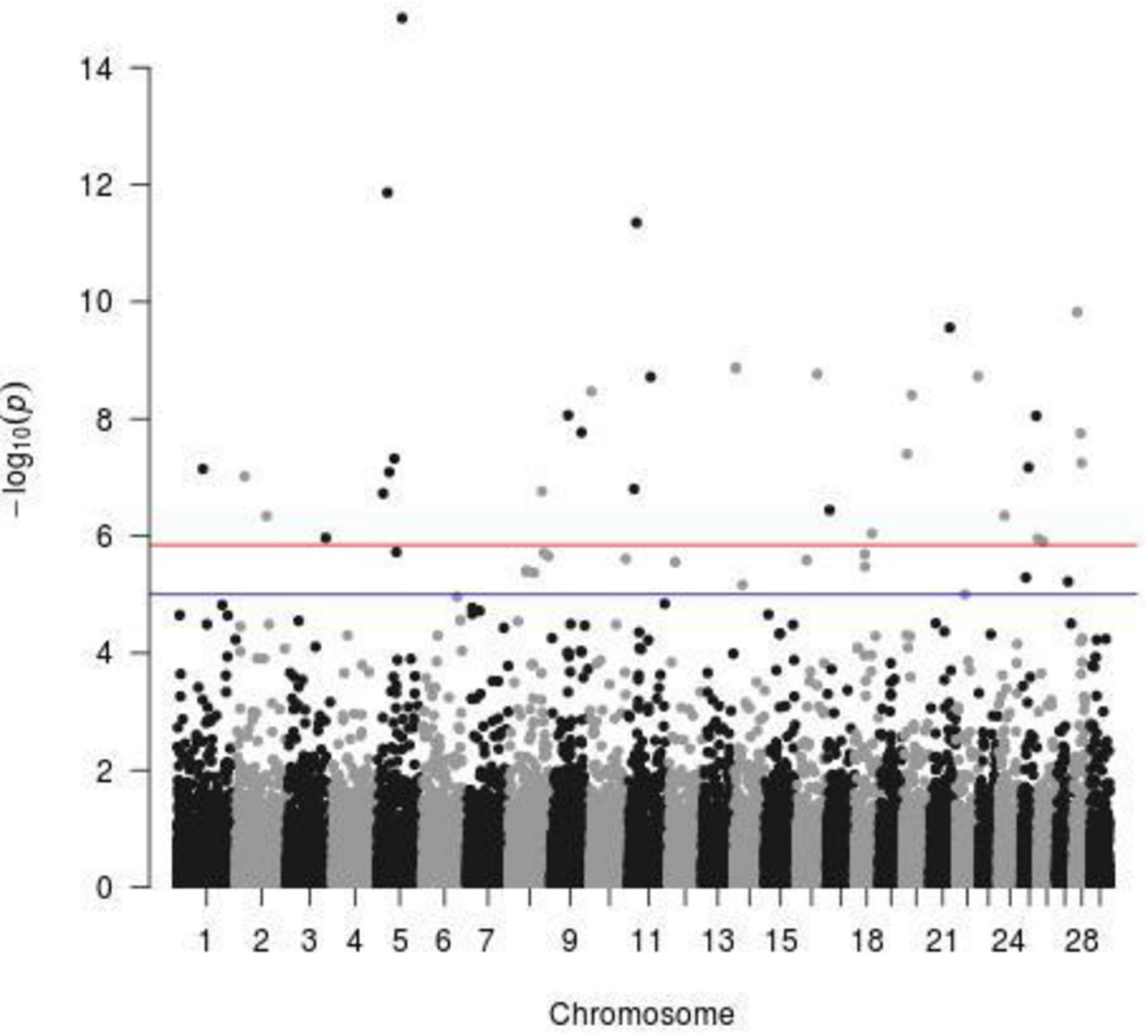
Significant SNPs for the ear position trait (blue line = indicative threshold–log10(p) = 5; red line = Bonferroni threshold–log10(p) = 5.86).

For dewlap size, there were multiple indicative but just two significant SNPs above the Bonferroni threshold (Fig 1). One was located on CHR 2 at 70.14Mb, with the surrounding genes CCDC93, INSIG2 and EN1, and the second on CHR 12 at 22.48Mb, with the surrounding genes LHFPL6, COG6 and FOXO1. As a possible candidate of an increased navel size, the HMGA2 gene on CHR 5 at 47.9Mb was identified in the Brasilian Nelore cattle [12]. BTA 5 has often been reported by studies that include Bos indicus cattle as crossbreds with Bos taurus, which is the case in our study; we can therefore say that this chromosome CHR5 carries genes that characterize both Zebu and Baoulé cattle. In Baoulé cattle, this chromosome CHR5 carries genes responsible for trypanotolerance [12] while in zebu the CHR5 carries genes related function in growth traits, and involved in immune response [13–15]. For our trait dewlap size, we did not identify any significant SNP in the region. Interestingly, for the ear position we found a significant SNP at 46.12 Mb in the immediate vicinity of HMGA2 (Fig 2). In general, for the ear position there were 30 significant SNPs above the Bonferroni threshold (Table 4), but none of them with a clear overlap with previously described ear morphology related genes [1].

**Table 4:** Significant SNP positions and genes detected for ear position.

CHR	Position (Mb)	p-value	Gene name
5	67.17	1.427976e-15	STAB2, NT5DC3, LOC505479, C5H12orf42, PAH
5	27.82	1.370694e-12	ACVR1B,FIGNL2,SCN8A,NR4A1,ATG101,KRT80,KRT7,KRT89, GRASP, ANKRD33
11	21.01	4.453821e-12	EPYC,DCN, LUM,KERA
28	10.24	1.501148e-10	RYR2
21	55.10	2.762805e-10	TP53BP1,ZSCAN29,MIS18BP1,TGM5,TOGARAM1,FRMD5,TUBGCP4,ADAL,PRPF39,FANCM,PPIP5K1
14	6.42	1.330248e-09	KHDRBS3
14	6.39	1.362059e-09	KHDRBS3
16	55.88	1.728063e-09	RABGAP1L,GPR52,TNN
22	59.30	1.869962e-09	EEFSEC,MGLL,KBTBD12,RUVBL1,GATA2, RAB7A,KIAA1257,IQSEC1,MGLL,GATA2,HMCES,RAB43,KIAA1257,EFCC1,IQSEC1
11	59.11	1.926522e-09	LRRTM4
10	3.51	3.379697e-09	KCNN2
20	24.24	3.920169e-09	LOC530348,SNX18,CDC20B, MTREX,DHX29,GZMK,LOC101908144
9	46.67	8.665168e-09	-
25	40.02	8.929194e-09	SDK1
9	82.80	1.701806e-08	EPM2A,SHPRH,FBXO30
28	20.63	1.770388e-08	-
20	11.47	3.997985e-08	PIK3R1,LOC101902212
5	46.12	4.76024e-08	DYRK2,CAND1
28	22.63	5.653865e-08	CTNNA3
25	19.17	6.763742e-08	LOC524391,LOC786628,CRYM,TMEM159,DNAH3,ANKS4B
1	69.10	7.167943e-08	KALRN,UMPS,ITGB5,MUC13,HEG1,SLC12A8
5	32.48	8.144792e-08	VDR,SENP1,PFKM,COL2A1,TMEM106C,RPAP3,RAPGEF3,HDAC7
2	22.77	9.539847e-08	CIR1,SP3,OLA1,GPR155, SCRN3,SP9,
11	14.39	1.576455e-07	MEMO1, SRD5A2,XDH,TGFA,SLC30A6,SPAST
8	89.04	8 1.72265e-07	SHC3,S1PR3
5	15.94	1.874109e-07	MGAT4C,RASSF9,NTS
17	7.83	3.639279e-07	DCLK2,IQCM
24	17.02	4.486478e-07	TRNAK-UUU
2	81.80	4.574848e-07	-
18	46.67	9.079853e-07	ZNF146,WDR62,NPHS1,NFKBID,ZNF382,KMT2B,ARHGAP33,APLP1,PROSER3,ATP4A,TBCB,ZNF461,ZNF529,ZNF565

### Genome-wide association for morphological traits—quantitative traits

The overview of the GWAS results for the quantitative traits is shown in Table 5. From these traits, height at withers and body length were measured for a higher number of individuals, as these were part of a general screening. The more detailed measurements were much more time intensive, therefore done on a smaller set, with number of measurements shown in Table 5.

**Table 5:** Genome wide significant SNPs effects for quantitative traits.

Traits	Number of chromosomes	Number of analyzed individuals
Height at withers	3, 8, 14	598
Body length	19	599
Chest width	2,5,8,9,11,14,16,20,21,25,26	198
Chest depth	3,5,7,11,14,15,16,18,23,	198
Chest girth	3,5,9,11,14,21	197
Head length	3,5,8,9,11,14,21,16,22,2528	192
Cranial length	1, 5, 7,8,11, 13, 16, 23	192
Head width	5,8,9,10,11,14,16,20,21,22,25,26,28	197
Cranial width	7	197
Earn length	1,2,5,6,8,11,14,20,22,28	194

The quantitative traits are by definition influenced by a large number of genes of small effect. The CHR5 carries genes related to the conformational traits of the African zebu breeds which are mostly kept for beef production. These genes are also in candidate region under selection in the Girolando zebu cattle from Asia, which has a large body size [16] Large scale GWAS analyses in humans uncovered hundreds of genes, which together explained about 20% of the variation in human height [17]. In our data set, even with the highest number of phenotypes we were not able to detect a large number of genes with plausible effects on height or size related traits.

The results were summarized in Table 6, showing overlaps between significant regions the between single trait analyses.

**Table 6:** Overlapping regions identified for quantitative trait GWAS analyses.

Chromosome	Position	Traits
5	27.823	Chest girth, head length, head width, ear length
	67.166	Chest width, head length, head width, chest girth
	109.513	Chest depth, cranial length
7	0.428	Cranial width, cranial length
	49.277	Chest depth, cranial length
8	89.046	Chest width, head width
9	46.669	Chest width, chest girth, head length, head width
	82.801	Chest width, head length
11	21.011	Earn length, chest width, head length, head width, chest girth
	21.146	Cranial length, chest depth
	59.110	Chest girth, chest depth, head length, head width, earn length
14	6.393	Head width, head length, chest girth, chest width
	6.416	Head width, head length, chest girth, chest width
16	55.882	Chest width, head length, head width
20	11.474	Head width, chest girth, chest width
21	55.096	Chest width, chest girth, head length, head width
22	59.306	Head length, earn length, head width
23	15.346	Chest depth, cranial length
25	19.174	Head width, chest width
26	14.934	Chest width, head width
28	10.241	Earn length, head width, head length

Note: The genomic regions were identified as ±0.5 Mb around the significant SNP

The significant region on CHR5 at 27.8 Mb contained a large number of genes, many related to keratin development, involved in the formation of hair [18], horns and wound repair [19]. In addition, ACVRL1 in the same genomic region is a growth factor, related to endothelia and blood vessel formation [20]. The relation of this genomic region to the traits, chest girth, head length, head width and ear length is not clear.

Another region on CHR5 at 67.166Mb harboured the most significant SNP nearby an unidentified pseudogene LOC112446821. Further nearby genes were STAB2, with a proven involvement in the bovine oviduct formation [21].

The region on CHR5 at 109.5 contained multiple genes of interest, the first one being the TRIOBP gene that was found to influence hearing impairment [22]. This gene could be of relevance to the cranial traits, although the proven effect seems to influence hair cell stereiocilia that is essential for hearing [23], rather than the cranial cavities. Another interesting gene in the same region was the ANKRD1 gene, influencing height at withers in horses [24].

The very beginning of CHR7 at around 0.5Mb contained a genomic region of interest, with two genes almost on the top of the most significant SNP. The FLT4 gene is involved in the proliferation and growth in cattle [25, 26]. Interestingly, it is also an endothelial and vascular growth factor similarly to the genes found on CHR5, and involved in the bovine oviduct formation [27].

The region on CHR7 at 49.3Mb harboured a fairly large number of genes. Among the relevant ones for the quantitative traits belongs the SPOCK1 gene, a growth factor [28], which appears to have influence on proteoglycans. Proteoglycans are a critical part of the extracellular structure, that act as a filler substance between the cells of the organism, but also act in a complex way to trap soluble growth factors in the hematopoetic cells and supportive tissues of the organism [29]. Another gene of great interest in the same region is EGR1 gene, a transcription factor with important roles in various cell types in response to different stimuli, in particular when it comes to the influence on bovine skeletal muscle via regulation of the MyoG gene expression [30].

The genomic region that was found as significant on CHR8 at 89.0 Mb did not contain any genes. The closest gene to this location was the MEF2C at 88.2 to 88.4 Mb with high relevance to growth. This gene, also known as Myocyte Enhancer Factor 2 plays a pivotal role in morphogenesis and myogenesis of skeletal, cardiac, and smooth muscle cells [31]. It was also shown to influence the growth of bovine muscle development in various stages of life in both Holstein and Limousin cattle [32]. An extremely interesting point is also the connection of the MEF2C gene to the development of the cardiovascular system [33]. Although, Xu et al. [33] highlights the importance of MEF2C in relation to retinal vessel loss, which is not closely related to the quantitative traits studied in this paper. This is the second occurrence along with the FLT4 gene on CHR7 when genes appear to have a pleiotropic effect related to development of the cardiovascular system and muscle growth. These findings put also the previously identified ACVRL1 gene on CHR5 into a new perspective. Although it was previously characterized only related to the cardiovascular system, we could hypothesize about its additional pleiotropic effects for growth following the same logic as for MEF2C and FLT4.

There were no genes of any kind located in the significant region at 46.7Mb on CHR9. The other region at CHR9 at 82.8Mb contained several genes such as UTRN, EPM2A, FBXO30, SHPRH and GRM1, but none of them with an apparent effect on quantitative size traits.

On CHR 11 the region around 21.2 Mb was found significant for a number of traits. This region harbors the GALM gene involved in glycol-metabolism and meat quality traits in cattle [34]. This genomic region was also identified to harbor a selection signature for body weight in Korean breeds [35]. The gene TMEM178A was found to be suggestively associated with loin muscularity in multiple beef cattle breeds [36]. In the same region was the MAP4K3 gene, involved in the Trypanosome resistance of the N’Dama cattle [37].

The region on CHR11 at 59.1Mb contained only LRRTM4, a single gene spanning about 1Mb in length. This gene was previously found to be associated with milk yield traits and mastitis resistance [38], as well as yearling weight in Hanwoo cattle [39].

The most significant SNP on CHR14 was directly on the top of the KHDRBS3 gene, found to be associated to fat deposition in cattle [26, 40], as well as cell growth and proliferation in a study of Brazilian Nelore cattle [41].

On CHR16 the most significant SNP at 55.8Mb was the nearest to the gene GPR52 with no apparent connection to size or growth traits, but it is involved in feed efficiency in *Bos indicus* beef cattle [42]. The CACYBP gene in the same region was related to skeletal muscle function in Holstein cattle [43].

The CHR20 contained a single region of interest at 11.5Mb, with PIK3R1 as the only protein coding gene, and the most significant SNP in our analysis on the top of it. The gene itself is involved in various cellular processes including cell survival, growth, proliferation and motility [44]. In cattle, it has been shown to be associated to milk production traits [45].

The significant region at CHR21 at 55.1Mb contained a lot of genes, but none of them had a plausible effect on size or growth traits that could be mentioned here.

The most significant SNP on CHR22 was at around 55.3Mb, containing the DNAJB8 gene previously identified as a heat shock protein gene in cattle [46]. Also the RUVBL1 gene nearby appears to contribute to thermos-tolerance mechanisms in African cattle [47]. The EEFSEC gene in the same region was identified having a pleiotropic effect in milk traits in Nordic Holstein (Cai et al., 2020) and amino acid concentration in Japanese Black beef cattle [48].

The top SNP on CHR23 was just nearby the FOXP4 gene at 15.3Mb. Although there is no specific information related to this gene in particular, the members of the FOXO gene family are known to be involved in numerous gene pathways, including growth in cattle [49]. The neighboring MDFI gene influences muscle development [50]. This part of the genome, with all genes around our most impactful SNP in our analysis was also highlighted as being positively selected towards Zebu genotypes in relation to Bovine leucocyte antigen (BoLA) region [51].

The most significant SNP on CHR25 at 19.2Mb is directly on the top of the unidentified protein coding ATP-binding cassette sub-family A member 3-like LOC524391 gene, with no further information available on it. The other genes in the region are DNAH3, ZP2 influencing fertility [52, 53] and feed intake [54] in cattle.

On CHR 26 at 14.9 Mb resides the MYOF gene witch has a proven effect on muscle biology [55]. Apart from this function, members of the ferlin family, of which Myoferyn is part of seem to regulate vascular endothelial growth, similarly to other genes highlighted in our current study. The RBP4 gene also from the same region was found to be involved in the insulin signaling pathways and glucose metabolism, with effects on growth and weight traits in cattle [56].

On CHR28 at 10.2Mb the most significant SNP was directly on top of a large gene RYR2, but his was without an apparent connection to growth traits or any other relevant aspect of the current study.

### Genome-wide association for trypanosome prevalence

**Fig 3:**
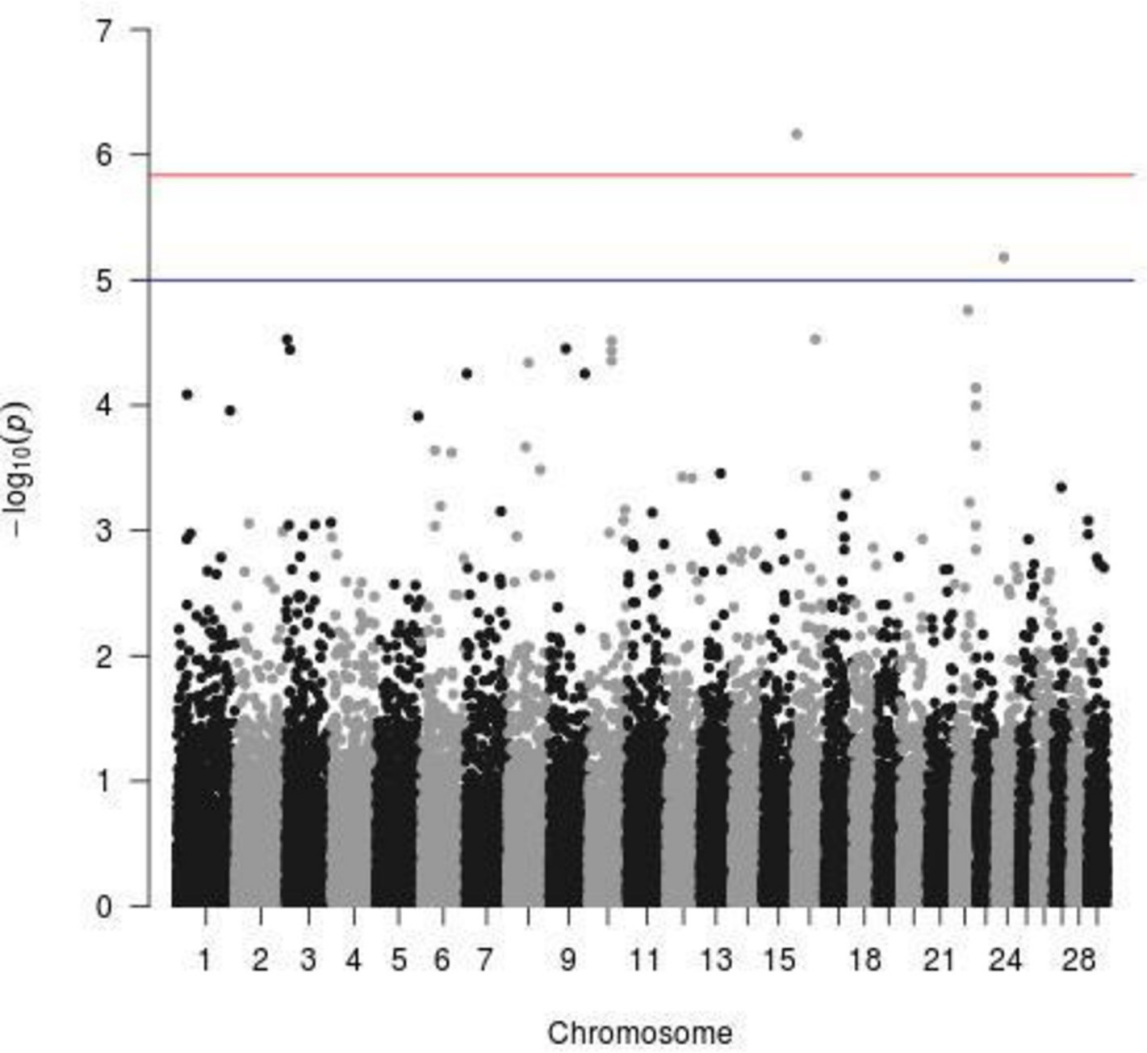
Significant SNPs related to resistance against trypanosomiasis in the purebred Baoulé cattle (blue line = indicative threshold–log10(p) = 5; red line = Bonferroni threshold–log10(p) = 5.86).

The results for genome-wide association for tolerance against trypanosomiasis in the purebred Baoulé cattle (Table 7) indicated two significant genomic regions (Fig 3). The first one was on CHR16 at 6.4Mb. Just 200kb downstream of our most significant SNP was the CFH gene. This gene was first identified in an epidemiology context in connection to malaria, where it acts as a negative regulator of complement to protect host tissues from aberrant complement activation [57]. Based on these functions, the CFH was selected as a candidate gene as it could produce protein products that are involved in the Human African Trypanosomiasis [58]. In the study of Tiberti et al. [59], the CFH was not found as a promising candidate, as it did not fulfil the arbitrary criteria to be selected in this study. Our results in the Baoulé cattle are closer to the findings of Ahouty et al. [60], who identified CFH as a candidate gene related to African Human Trypanosomiasis. The other of the two protein coding genes in the same region was KCNT2, which does not seem to be relevant for trypanotolerance, although it was found to be connected to other disorders, such as ketosis [61] and metritis [62] in Holstein cattle. The CHR16 (23.10–23.40 Mb) has also been identified by Smetko et al. [63] as potentially implicated in trypanotolerance, which is confirmed in our study.

**Table 7:** Chromosomes with significant effects for trypanosomosis status.

Trypanosomosis status	Chromosome number	Number of analyzed individuals
Purebred Baoulé	16,24	332
Crossbreds	5	278

The second significant region for trypanotolerance in our purebred Baoulé cattle data set was on CHR24 at 22.5Mb. The most significant SNP was on the top of the LRRN1 gene, which was not found to be in a direct connection to trypanosomiasis. The CRBN gene however at around 23.1Mb, so around 600kb upstream from our signal is a more promising candidate. The CRBN gene forms the Cereblon protein, and its orthologs are highly conserved from plants to humans, which underscores its physiological importance. Cereblon forms an E3 ubiquitin ligase complex, which in turn was shown to have parasite-specific effects, in particular in connection to malaria [64]. This pattern of an effect related to parasite and malaria are reminiscent of the CFH gene as discussed above. Along the same lines, we hypothesize that this gene could be an additional candidate for trypanosomiasis resistance that was not highlighted in any of the previous studies. The two additional nearby genes TRNT1 and IL5RA also seem to be involved in immunological processes [65–67] which could be also relevant for the trypanosomiasis resistance in cattle.

**Fig 4:**
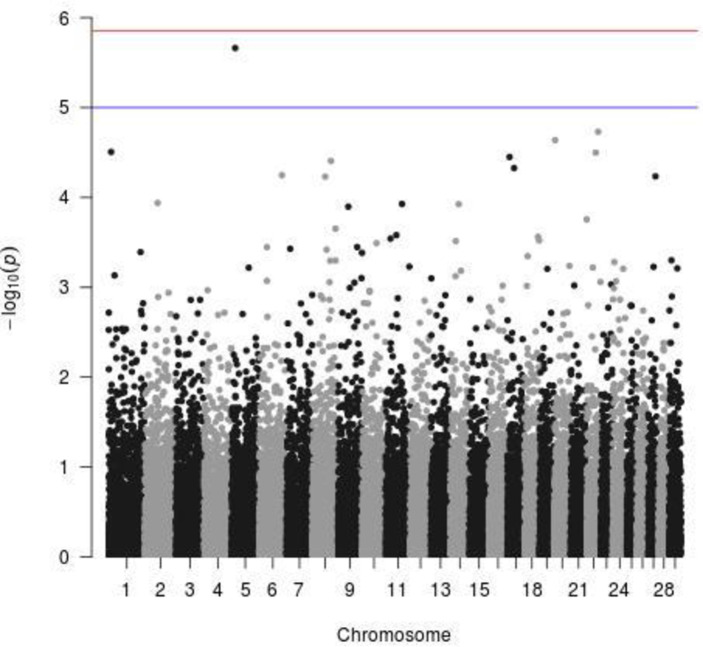
Significant SNPs related to tolerance against trypanosomiasis in crossbred cattle (blue line = indicative threshold–log10(p) = 5; red line = Bonferroni threshold–log10(p) = 5.86).

In crossbreds, the most significant SNP was in a gene sparse region of CHR5 at 16.1 Mb (Fig 4). The closest gene to the most significant SNP was the MGAT4C about 150kb downstream, which was found to affect the growth rate in buffaloes [68], seemingly not in direct connection to trypanosomiasis. An indirect lead to its connection to trypanosomiasis resistance could be that the MGAT4C gene was also identified as a QTL for Cytokynes [69], which are proteins produced by T-lymphocytes as an immunological response to viral and non-viral antigens.

The second gene about 600kb downstream of the most significant SNP is NTS (Neurotensin), whose degradation is mediated by the enzyme of the *T*. *brucei* prolyl oligopeptidase gene, providing a clear connection to trypanosomiasis and the sleeping sickness disease that it causes. The POP Tb enzyme of the *T*. *brucei* might not completely deactivate the peptides, as NTS generated products with weaker affinity for their receptors [70]. A similar effect was discussed also in Morty et al. [71] about the influence of peptidases released by trypanosomes that could affect regulatory peptides such as neurotensin and gastrin. Interestingly, the other genes related to trypanosomiasis, identified in our study (TRNT1 and IL5RA) were also connected to immunological response.

Although the regions identified in our study seem to be relevant to the trypanotolerance, we did not find an overlap between our results and some of the notable previous studies. In N’Dama cattle, Hanotte et al. [72] mapped QTL associated with trypanotolerance on 18 different cattle autosomes in F2 crosses of trypanotolerant N’Dama and trypanosusceptible Boran cattle. They showed independent genetic control for parasitaemia and body weight, with most QTLs having minor effects and the major ones being located on chromosomes 2, 4, 7, 16 and 27. Subsequently, expression analyses in blood cells reported pathways and genes differentially regulated in trypanotolerant N’Dama and trypanosusceptible Kenyan Boran [73, 74]. Genes such as *TICAM1*, *ARHGAP15*, *SLC40A1*, *GFM1* and *INHBA* have been proposed as candidate genes for trypanotolerance [7, 74]. More recently, full genome sequence analysis reported several candidate genome regions under positive selection in N’Dama cattle including genes with functions related to immunity, anaemia and feeding behaviours that may be linked to the trypanotolerant phenotypes [47, 75]. Tijjani [12] considered the common candidate genes in Muturu and N’Dama breeds and reported pathways linked to trypanotolerance in West African taurine population as well as selected candidate genes in Muturu cattle only. In total, the authors identified 20 candidate genes in West African taurines. Functional annotation and enrichment analyses based on Reactome pathways in PANTHER *ver13*.*1* [76] confirmed their relevance in response to trypanosome infection pathways. They are all members of the major histocompatibility complex (MHC) class II with related functions in immune responses. Although our results also point into the direction of genes involved in immunological response, we did not find exact overlaps with regions identified in Tijjani, [12] and Hanotte et al. [72].

## Conclusions

Overall, our study allowed us to identify genes potentially responsible for the morphological traits in Baoulé cattle and their crossbreds. GWAS analysis identified several putative candidate regions spanning genes relating to morphology and trypanosome tolerance. We have attempted to identify candidate genes responsible for the distinguishing features between the taurine and zebu cattle based on dewlap size and ear shape, but we did not find any apparent genomic region of interest.

This study also includes GWAS characteristics of 25 quantitative, morphological traits. Although we did not identify genes that could be related directly to size traits, many of the genes were previously highlighted in various growth-related studies. A re-occurring theme for the several genes residing in the regions identified by the most significant SNPs was pleiotropic effect on growth of the body and the cardiovascular system.

The results for genome-wide association for trypanotolerance in the purebred Baoulé and their crossbreds indicated three significant regions on CHR16 and CHR24 in purebred Baoulé, and on CHR5 in Baoulé x Zebu crossbreds, all of which contained genes previously reported to be relevant to trypanotolerance. Among these, perhaps the most important finding was the CRBN gene, which, to the best of our knowledge has not been connected with trypanosomiasis before. Additional genes highlighted in our study were CFH, TRNT1, IL5RA, MGAT4C and NTS, which could be also relevant for the trypanosomiasis resistance in cattle.

## Supporting information

S1 Fig Significant SNPs for the chest width trait (blue line = indicative threshold–log10(p) = 5; red line = Bonferroni threshold–log10(p) = 5.86). (DOCX)

S2 Fig Significant SNPs for the chest depth trait (blue line = indicative threshold–log10(p) = 5; red line = Bonferroni threshold–log10(p) = 5.86). (DOCX)

S3 Fig Significant SNPs for the chest girth trait (blue line = indicative threshold–log10(p) = 5; red line = Bonferroni threshold–log10(p) = 5.86). (DOCX)

S4 Fig Significant SNPs for the head length trait (blue line = indicative threshold–log10(p) = 5; red line = Bonferroni threshold–log10(p) = 5.86). (DOCX)

S5 Fig Significant SNPs for the cranial length trait (blue line = indicative threshold–log10(p) = 5; red line = Bonferroni threshold–log10(p) = 5.86). (DOCX)

S6 Fig Significant SNPs for the head width trait (blue line = indicative threshold–log10(p) = 5; red line = Bonferroni threshold–log10(p) = 5.86). (DOCX)

S7 Fig Significant SNPs for the cranial width trait (blue line = indicative threshold–log10(p) = 5; red line = Bonferroni threshold–log10(p) = 5.86). (DOCX)

S8 Fig Significant SNPs for the earn length trait (blue line = indicative threshold–log10(p) = 5; red line = Bonferroni threshold–log10(p) = 5.86). (DOCX)

S1 Table Significant SNP positions and genes detected for chest width. (DOCX)

S2 Table Significant SNP positions and genes detected for chest depth. (DOCX)

S3 Table Significant SNP positions and genes detected for chest girth. (DOCX)

S4 Table Significant SNP positions and genes detected for head length. (DOCX)

S5 Table Significant SNP positions and genes detected for cranial length. (DOCX)

S6 Table Significant SNP positions and genes detected for head width. (DOCX)

S7 Table Significant SNP positions and genes detected for cranial width. (DOCX)

S8 Table Significant SNP positions and genes detected for earn length. (DOCX)

S1 File Genotype _data_cattle-Burkina Faso. (ZIP)

S2 File Morphometrics_data_cattle-Burkina Faso. (ZIP)
