# Supplementary material for: Genome-wide association study of trypanosome prevalence and morphometric traits in purebred and crossbred Baoulé cattle of Burkina Faso
Source: PLoS One. 2021 Aug 5;16(8):e0255089. doi: 10.1371/journal.pone.0255089 (PMC8341487; doi:10.1371/journal.pone.0255089)
Supplement: S6 Fig — (DOCX) [file pone.0255089.s006.docx]

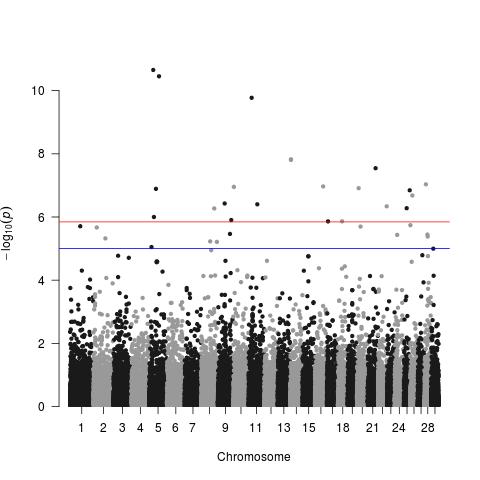


**S6 Fig.** Significant SNPs for the Head width trait (blue line = indicative threshold –log10(p) = 5; red line = Bonferroni threshold –log10(p) = 5.86)
