# Supplementary material for: Genome-wide association study of trypanosome prevalence and morphometric traits in purebred and crossbred Baoulé cattle of Burkina Faso
Source: PLoS One. 2021 Aug 5;16(8):e0255089. doi: 10.1371/journal.pone.0255089 (PMC8341487; doi:10.1371/journal.pone.0255089)
Supplement: S1 Table — (DOCX) [file pone.0255089.s009.docx]

**S1 Table.** Significant SNP positions and genes detected for chest width

| Chromosome | Name | Position (bp) | p-value | Gene name |
| --- | --- | --- | --- | --- |
| 5 | ARS-USDA-AGIL-chr5-27822665-000674 | 27822665 | 8.498384e-12 | SCN8A, ACVR1B, NR4A1, KRT80, KRT89, KRT7, FIGNL2, ATG101 |
| 5 | BovineHD0500018790 | 67166089 | 4.779147e-11 | STAB2,C5H12orf42,PAH,LOC505479,NT5DC3, |
| 21 | ARS-BFGL-NGS-43284 | 55096333 | 5.713037e-10 | TP53BP1, TUBGCP4, ADAL, MIS18BP1, TGM5, TOGARAM1, FANCM, FRMD5, PPIP5K1 |
| 11 | BovineHD1100006313 | 21010994 | 5.891361e-10 | GALM, HNRNPLL, SOS1, DHX57, ARHGEF33, GEMIN6, |
| 25 | BovineHD4100017514 | 40017330 | 4.448551e-09 | SDK1,CARD11,MIR2390,LOC112444323,LOC107131851 |
| 20 | BovineHD2000003694 | 11473987 | 3.760484e-08 | PIK3R1, LOC101902212 |
| 5 | BovineHD0500013248 | 46123693 | 4.318377e-08 | DYRK2, CAND1 |
| 26 | ARS-BFGL-NGS-28793 | 14934474 | 7.30534e-08 | ABCC1, ABCC6, LOC515570, LOC100296255 |
| 8 | BovineHD0800026422 | 89046034 | 8.172296e-08 | SHC3, S1PR3 |
| 14 | ARS-BFGL-NGS-43719 | 6415535 | 1.70845e-07 | KHDRBS3 |
| 14 | BovineHD1400001697 | 6393247 | 1.76427e-07 | KHDRBS3 |
| 2 | Hapmap33788-BTA-154116 | 81796035 | 2.642213e-07 | - |
| 9 | BovineHD0900012890 | 46668612 | 6.193228e-07 | LOC112448167 |
| 16 | BovineHD1600015547 | 55882084 | 7.478795e-07 | RABGAP1L, TNN, GPR52 |
| 25 | ARS-BFGL-NGS-23517 | 19173921 | 9.526397e-07 | LOC524391,LOC786628,CRYM,TMEM159,DNAH3,ANKS4B |
| 9 | BovineHD0900023037 | 82800536 | 9.899314e-07 | EPM2A, SHPRH, FBXO30 |
