# Supplementary material for: Genome-wide association study of trypanosome prevalence and morphometric traits in purebred and crossbred Baoulé cattle of Burkina Faso
Source: PLoS One. 2021 Aug 5;16(8):e0255089. doi: 10.1371/journal.pone.0255089 (PMC8341487; doi:10.1371/journal.pone.0255089)
Supplement: S2 Table — (DOCX) [file pone.0255089.s010.docx]

**S2 Table.** Significant SNP positions and genes detected for chest deph

| Chromosome | Name | Position (bp) | P-value | Gene name |
| --- | --- | --- | --- | --- |
| 5 | BovineHD0500031566 | 109513108 | 3.416984e-11 | TRIOBP,GGA1, MICAL3,SH3BP1, PLA2G6, MICALL1, BAIAP2L2 |
| 15 | ARS-BFGL-NGS-23895 | 75178820 | 1.037945e-09 | PRDM11, TSPAN18, TP53I11, SYT13 |
| 11 | BovineHD1100006344 | 21146006 | 1.278198e-09 | DHX57, ARHGEF33, SOS1,CDKL4, MAP4K3,GALM, ATL2,GEMIN6, HNRNPLL |
| 16 | BovineHD1600017047 | 60707911 | 3.439376e-09 | AXDND1, TDRD5,SOAT1, ABL2, FAM163A, RALGPS2, FAM20B, TOR1AIP2,NPHS2 |
| 3 | Hapmap48592-BTA-107734 | 73687938 | 2.874174e-08 | NEGR1,ZRANB2 |
| 5 | BovineHD0500018790 | 67166089 | 5.329412e-08 | STAB2, C5H12orf42, LOC505479 |
| 7 | BovineHD0700014288 | 49277199 | 1.180413e-07 | KLHL3,SPOCK1, FAM13B, PKD2L2,MYOT |
| 23 | ARS-BFGL-NGS-5042 | 15345878 | 1.410175e-07 | CCND3,FOXP4, TFEB, USP49, TREM2,TREM1 |
| 18 | BovineHD1800001365 | 4661819 | 3.360002e-07 | ADAMTS18, SYCE1L,NUDT7, VAT1L,MON1B |
| 14 | UA-IFASA-8629 | 41836247 | 4.418524e-07 | PKIA,ZC2HC1A, IL7 |
| 11 | ARS-BFGL-NGS-82127 | 59110486 | 5.72765e-07 | LRRTM4 |
