# Supplementary material for: Genome-wide association study of trypanosome prevalence and morphometric traits in purebred and crossbred Baoulé cattle of Burkina Faso
Source: PLoS One. 2021 Aug 5;16(8):e0255089. doi: 10.1371/journal.pone.0255089 (PMC8341487; doi:10.1371/journal.pone.0255089)
Supplement: S3 Table — (DOCX) [file pone.0255089.s011.docx]

**S3 Table.** Significant SNP positions and genes detected for chest girth

| Chromosome | Name | Position(bp) | P-value | Gene name |
| --- | --- | --- | --- | --- |
| 5 | BovineHD0500018790 | 67166089 | 6.641699e-13 | STAB2,C5H12orf42, LOC505479,NT5DC3, PAH |
| 11 | ARS-BFGL-NGS-82127 | 59110486 | 8.698211e-11 | LRRTM4 |
| 21 | ARS-BFGL-NGS-43284 | 55096333 | 6.626392e-09 | MIS18BP1, TOGARAM1,FANCM,TGM5,ADAL, TP53BP1,FRMD5, TUBGCP4,WDR76, PPIP5K1 |
| 5 | ARS-USDA-AGIL-chr5-27822665-000674 | 27822665 | 8.426185e-09 | SCN8A,ACVR1B, NR4A1,KRT80, FIGNL2 |
| 11 | BovineHD1100006313 | 21010994 | 2.977185e-08 | GALM,GEMIN6, DHX57,ARHGEF33, SOS1,ATL2 |
| 16 | BovineHD1600015547 | 55882084 | 4.394731e-08 | RABGAP1L,TNN, GPR52,MRPS14 |
| 14 | ARS-BFGL-NGS-43719 | 6415535 | 1.487558e-07 | KHDRBS3 |
| 14 | BovineHD1400001697 | 6393247 | 1.616635e-07 | KHDRBS3 |
| 9 | BovineHD0900012890 | 46668612 | 2.933311e-07 | LOC112448167 |
| 3 | ARS-BFGL-NGS-118243 | 104909909 | 3.174244e-07 | SCMH1,HIVEP3, FOXO6,CTPS1,SLFNL1, EDN2 |
| 20 | BovineHD2000003694 | 11473987 | 6.950299e-07 | PIK3R1 |
