# Supplementary material for: Genome-wide association study of trypanosome prevalence and morphometric traits in purebred and crossbred Baoulé cattle of Burkina Faso
Source: PLoS One. 2021 Aug 5;16(8):e0255089. doi: 10.1371/journal.pone.0255089 (PMC8341487; doi:10.1371/journal.pone.0255089)
Supplement: S4 Table — (DOCX) [file pone.0255089.s012.docx]

**S4 Table.** Significant SNP positions and genes detected for head length

| Chromosome | Name | Position (bp) | P-value | Gene name |
| --- | --- | --- | --- | --- |
| 5 | BovineHD0500018790 | 67166089 | 4.608976e-14 | STAB2,NT5DC3, C5H12orf42, PAH |
| 5 | ARS-USDA-AGIL | 27822665 | 3.967678e-10 | SCN8A, ACVR1B, KRT80, NR4A1, |
| 11 | BovineHD1100006313 | 21010994 | 1.400638e-09 | GALM,GEMIN6, DHX57, ARHGEF33, SOS1, ATL2 |
| 21 | ARS-BFGL-NGS -43284 | 55096333 | 2.876252e-09 | TP53BP1, TUBGCP4, MIS18BP1, TGM5, TOGARAM1 |
| 16 | BovineHD1600015547 | 55882084 | 5.689687e-09 | RABGAP1L,TNN, GPR52, MRPS14 |
| 14 | ARS-BFGL-NGS-43719 | 6415535 | 7.118057e-09 | KHDRBS3 |
| 14 | BovineHD1400001697 | 6393247 | 8.092584e-09 | KHDRBS3 |
| 11 | ARS-BFGL-NGS-82127 | 59110486 | 1.333776e-08 | LRRTM4 |
| 28 | BovineHD2800003136 | 10240554 | 6.473525e-08 | RYR2 |
| 25 | BovineHD4100017514 | 40017330 | 1.140717e-07 | SDK1, CARD11 |
| 9 | BovineHD0900023037 | 82800536 | 2.324168e-07 | EPM2A, SHPRH,FBXO30, |
| 22 | BovineHD2200017232 | 59306048 | 3.58011e-07 | EEFSEC,MGLL, RAB7A,KBTBD12, RUVBL1, KIAA1257, ACAD9 |
| 8 | BovineHD0800031870 | 106690894 | 4.025093e-07 | ASTN2 |
| 3 | ARS-BFGL-NGS-118243 | 104909909 | 4.267078e-07 | SCMH1,HIVEP3, FOXO6,CTPS1, EDN2 |
| 9 | BovineHD0900012890 | 46668612 | 4.402174e-07 | - |
