# Supplementary material for: Genome-wide association study of trypanosome prevalence and morphometric traits in purebred and crossbred Baoulé cattle of Burkina Faso
Source: PLoS One. 2021 Aug 5;16(8):e0255089. doi: 10.1371/journal.pone.0255089 (PMC8341487; doi:10.1371/journal.pone.0255089)
Supplement: S5 Table — (DOCX) [file pone.0255089.s013.docx]

**S5 Table.** Significant SNP positions and genes detected for cranial length

| Chromosome | Name | Position (bp) | P-value | Gene name |
| --- | --- | --- | --- | --- |
| 23 | ARS-BFGL-NGS-5042 | 15345878 | 2.222193e-10 | FOXP4,MDFI, TFEB,USP49CCND3, USP49,TREM2 |
| 5 | BovineHD0500031566 | 109513108 | 2.378191e-10 | TRIOBP, PLA2G6,  MICAL3, MICALL1, GGA1, BAIAP2L2 |
| 11 | BovineHD1100006344 | 21146006 | 4.501103e-10 | DHX57,SOS1, CDKL4, ARHGEF33, GEMIN6, GALM |
| 7 | BovineHD0700014288 | 49277199 | 3.958158e-08 | FAM13B, KLHL3,PKD2L2, SPOCK1,MYOT, HNRNPA0 |
| 16 | BovineHD1600017047 | 60707911 | 1.673641e-07 | AXDND1,SOAT1, TDRD5,FAM163A, ABL2,NPHS2, TOR3A |
| 1 | Hapmap50953-BTA-39491 | 9375752 | 2.703693e-07 | ADAMTS5, ADAMTS1 |
| 13 | Hapmap47254-BTA-33890 | 77287986 | 2.894847e-07 | STAU1,PREX1, KCNB1,CSE1L, ARFGEF2, PTGIS |
| 7 | ARS-BFGL-NGS-19919 | 428335 | 4.017933e-07 | FLT4,CNOT6, MAPK9, GFPT2 |
| 8 | ARS-USDA-AGIL-chr8-85820133-000130 | 85820133 | 9.340231e-07 | - |
