# Supplementary material for: Genome-wide association study of trypanosome prevalence and morphometric traits in purebred and crossbred Baoulé cattle of Burkina Faso
Source: PLoS One. 2021 Aug 5;16(8):e0255089. doi: 10.1371/journal.pone.0255089 (PMC8341487; doi:10.1371/journal.pone.0255089)
Supplement: S6 Table — (DOCX) [file pone.0255089.s014.docx]

**S6 Table.** Significant SNP positions and genes detected for head width

| Chromosome | Name | Position (bp) | P-value | Gene name |
| --- | --- | --- | --- | --- |
| 5 | ARS-USDA-AGIL-chr5-27822665-000674 | 27822665 | 2.217103e-11 | SCN8A,ACVR1B,FIGNL2,ATG101,NR4A1,KRT80 |
| 5 | BovineHD1100006313 | 67166089 | 3.521616e-11 | STAB2, C5H12orf42, NT5DC3 |
| 11 | BovineHD1100006313 | 21010994 | 1.68848e-10 | HNRNPLL,GALM,ATL2,GEMIN6,DHX57ARHGEF33,SOS1 |
| 14 | BovineHD1400001697 | 6393247 | 1.49256e-08 | KHDRBS3 |
| 14 | ARS-BFGL-NGS-43719 | 6415535 | 1.556848e-08 | KHDRBS3 |
| 21 | ARS-BFGL-NGS-43284 | 55096333 | 2.851781e-08 | TP53BP1,ZSCAN29,MIS18BP1,TGM5,TOGARAM1,FRMD5,TUBGCP,ADAL,PRPF39,FANCM,PPIP5K1 |
| 28 | BovineHD2800003136 | 10240554 | 9.238275e-08 | RYR2 |
| 16 | BovineHD1600015547 | 55882084 | 1.07665e-07 | RABGAP1L,TNN,MRPS14 |
| 10 | BovineHD1000001079 | 3508025 | 1.109316e-07 | KCNN2 |
| 20 | BovineHD2000003694 | 11473987 | 1.221809e-07 | STYX,SERINC5,TXNDC16,GPR137C,ERO1A,PSMC6 |
| 5 | BovineHD0500013248 | 46123693 | 1.281221e-07 | DYRK2,CAND1 |
| 25 | BovineHD4100017514 | 40017330 | 1.424275e-07 | SDK1 |
| 26 | ARS-BFGL-NGS-28793 | 14934474 | 2.084658e-07 | FRA10AC1, MYOF, CEP55, FFAR4, PDE6C, RBP4, LGI1, SLC35G1 |
| 9 | BovineHD0900012890 | 46668612 | 3.737301e-07 | - |
| 11 | ARS-BFGL-NGS-82127 | 59110486 | 3.960296e-07 | LRRTM4 |
| 22 | BovineHD2200017232 | 59306048 | 4.593525e-07 | RAB7A,EEFSEC, MGLL, KBTBD12, RUVBL1, GATA2, HMCES, RAB43, KIAA1257, EFCC1,IQSEC1 |
| 25 | ARS-BFGL-NGS-23517 | 19173921 | 5.268129e-07 | LOC524391, TMEM159, CRYM,  LOC786628,DNAH3,ANKS4B |
| 8 | BovineHD0800026422 | 89046034 | 5.352788e-07 | SHC3, S1PR3 |
| 5 | BovineHD4100003572 | 32481519 | 9.997703e-07 | - |
