# Supplementary material for: Genome-wide association study of trypanosome prevalence and morphometric traits in purebred and crossbred Baoulé cattle of Burkina Faso
Source: PLoS One. 2021 Aug 5;16(8):e0255089. doi: 10.1371/journal.pone.0255089 (PMC8341487; doi:10.1371/journal.pone.0255089)
Supplement: S7 Table — (DOCX) [file pone.0255089.s015.docx]

**S7 Table.** Significant SNP positions and genes detected for cranial width

| Chromosome | Name | Position (bp) | P-value | Gene name |
| --- | --- | --- | --- | --- |
| 7 | chr7_36055243 | 36055243 | 1.205233e-06 | LOC112447397 |
| 15 | BovineHD1500002422 | 9327786 | 1.594589e-06 | CNTN5 |
| 7 | ARS-BFGL-NGS-19919 | 428335 | 2.090888e-06 | FLT4,CNOT6,MAPK9,GFPT2 |
| 8 | BovineHD0800007630 | 25238250 | 2.245373e-06 | SAXO1,HAUS6,ADAMTSL1 |
