# Supplementary material for: Genome-wide association study of trypanosome prevalence and morphometric traits in purebred and crossbred Baoulé cattle of Burkina Faso
Source: PLoS One. 2021 Aug 5;16(8):e0255089. doi: 10.1371/journal.pone.0255089 (PMC8341487; doi:10.1371/journal.pone.0255089)
Supplement: S8 Table — (DOCX) [file pone.0255089.s016.docx]

**S8 Table.** Significant SNP positions and genes detected for earn length

| Chromosome | Name | Position (bp) | P-value | Gene name |
| --- | --- | --- | --- | --- |
| 5 | ARS-USDA-AGIL-chr5-27822665-000674 | 27822665 | 1.076894e-10 | SCN8A,ACVR1B,FIGNL2,ATG101,NR4A1,KRT80 |
| 11 | ARS-BFGL-NGS-82127 | 59110486 | 1.409039e-10 | LRRTM4 |
| 5 | BovineHD0500018790 | 67166089 | 3.288332e-10 | ANTXR1,ARHGAP25,APLF,PROKR1,LOC509961,PLEK |
| 20 | BovineHD2000007299 | 24237964 | 4.147857e-09 | LOC530348,CDC20B,MTREX,SNX18,DHX29,ESM1,GZMK |
| 11 | BovineHD1100006313 | 21010994 | 8.34563e-09 | HNRNPLL,GALM,ATL2,GEMIN6,DHX57,ARHGEF33,SOS1 |
| 22 | BovineHD2200017232 | 59306048 | 8.785567e-09 | EEFSEC,MGLL, KBTBD12, RUVBL1, GATA2,RAB7A,KIAA1257,IQSEC1,MGLL,GATA2,HMCES,RAB43,KIAA1257,EFCC1,IQSEC1 |
| 5 | BovineHD4100003572 | 32481519 | 2.061277e-08 | VDR,SENP1,PFKM,TMEM106C,COL2A1,RPAP3,RAPGEF3,HDAC7,ENDOU |
| 28 | BTB-00980887 | 22633575 | 2.380179e-08 | CTNNA3 |
| 2 | BovineHD0200006467 | 22767068 | 3.247986e-08 | OLA1, SP3,SCRN3, GPR155,CIR1 |
| 14 | BovineHD1400007035 | 24263980 | 3.330059e-08 | FAM110B,IMPAD1,SDCBP,CYP7A1,UBXN2B |
| 6 | Hapmap25280-BTA-144335 | 98788966 | 3.98234e-08 | GPAT3 |
| 6 | BovineHD0600002391 | 10089189 | 4.207174e-08 | NDST4 |
| 8 | BovineHD0800014142 | 47218664 | 1.228517e-07 | - |
| 8 | BovineHD0800033706 | 47305437 | 1.228517e-07 | _ |
| 1 | BovineHD0100019566 | 69097650 | 2.919156e-07 | KALRN,UMPS,ITGB5,HEG1,MUC13,SLC12A8 |
| 1 | BovineHD0100037350 | 131373643 | 3.706875e-07 | CLDN18,DZIP1L,ARMC8,SOX14,LOC107132214,A4GNT,DBR1 |
| 5 | BovineHD0500013248 | 46123693 | 4.840572e-07 | DYRK2,CAND1 |
| 27 | BovineHD2700008776 | 31424496 | 5.552966e-07 | UNC5D,LOC112444609 |
| 28 | BovineHD2800003136 | 10240554 | 5.616433e-07 | RYR2, ZP4 |
| 5 | Hapmap40277-BTA-72811 | 15939304 | 6.186923e-07 | MGAT4C,RASSF9,NTS |
